# Promoting Oral Health Literacy Among UAE Public Sector Employees

**DOI:** 10.2196/66452

**Published:** 2024-11-12

**Authors:** Satish C Nair

**Affiliations:** 1 College of Medicine, UAE University and Tawam Hospital, Tawam Street, Al Ain, 15258, United Arab Emirates, 971 0547591003

**Keywords:** health literacy, Gulf countries, oral health literacy, health promotion, United Arab Emirates, workplace

The recent publication “Promoting Health Literacy in the Workplace Among Civil Servants: Cross-Sectional Study” by Carrouel et al, in *JMIR Public Health & Surveillance*, is strikingly intriguing [1]. Harnessing the potential of health promotion, particularly health literacy in the workplace, is clever, given the fact that more and more employees spend a huge proportion of their daily hours at their workplace. Poor oral health can result in psychological distress because of appearance and also affect the daily requirement for nutrients, given the low food intake by patients with oral diseases. Additionally, periodontitis has been correlated with the risk for cardiovascular disease and hypertension, and it reportedly possesses a bidirectional relationship with diabetes [2].

The study’s results hold promise because none of the French civil servants surveyed had inadequate oral health literacy levels, although approximately 10% had marginal levels of oral health knowledge and less than 20% had marginal oral health literacy [1]. Interestingly, it has been reported that children whose parents possessed low oral health literacy demonstrated toxic oral health behavior. Low oral health literacy among parents correlated with a higher prevalence of dental caries and an increased number of tooth fillings and extractions among their children [3]. In the Middle East, especially among the Gulf Cooperation Council countries, the burden of overall oral disease is increasing given the inadequate knowledge of oral health literacy among parents. Children in the United Arab Emirates have been reported to consume sugar-rich flavoured drinks increasingly and have a high carbohydrate diet. UAE nationals are covered for oral health under the national insurance policy, but the vast majority of expatriates, almost 9 million, lack oral health insurance coverage. This is particularly alarming in a country where the prevalence of diabetes is approximately 19%, and cardiovascular diseases are on the rise [4,5]. A nationwide study from the United Arab Emirates indicated an overall 11% health literacy level among adult patients with type 2 diabetes [5]. Taking a cue from the survey of French civil servants, the United Arab Emirates should explore a model of oral health literacy promotion among their public sector employees. The estimation of oral health literacy as a module of pre-employment screening for public sector jobs in the United Arab Emirates should be easy to implement given the successful adoption of digital technology by the UAE government. Subsequently, a national registry of oral health literacy for the UAE population will be useful to trace, track, and treat oral health diseases in the country, to prevent a health crisis.
